# How to direct patients to high-volume hospitals: exploring the influencing drivers

**DOI:** 10.1186/s12913-023-10229-9

**Published:** 2023-11-16

**Authors:** Elisabetta Listorti, Erica Pastore, Arianna Alfieri

**Affiliations:** 1 Centre for Research on Health and Social Care Management (CERGAS), SDA Bocconi School of Management - Bocconi University, Milan, Italy; 2 Department of Management and Production Engineering, Politecnico di Torino, Turin, Italy

**Keywords:** Patients, Volume-outcome association, Volume of activity, Colon Cancer, Regional hospital planning, Choice, Logistic regression

## Abstract

**Background:**

During the last decade, planning concentration policies have been applied in healthcare systems. Among them, attention has been given to guiding patients towards high-volume hospitals that perform better, acccording to the *volume-outcome* association. This paper analyses which factors drive patients to choose big or small hospitals (with respect to the international standards of volumes of activity).

**Methods:**

We examined colon cancer surgeries performed in Piedmont (Italy) between 2004 and 2018. We categorised the patient choice of the hospital as big/small, and we used this outcome as main dependent variable of descriptive statistics, tests and logistic regression models. As independent variables, we included (i) patient characteristics, (ii) characteristics of the closest big hospital (which should be perceived as the most immediate to be chosen), and (iii) territorial characteristics (i.e., characteristics of the set of hospitals among which the patient can choose). We also considered interactions among variables to examine which factors influence all or a subset of patients.

**Results:**

Our results confirm that patient personal characteristics (such as age) and hospital characteristics (such as distance) play a primary role in the patient decision process. The findings seem to support the importance of closing small hospitals when they are close to big hospitals, although differences emerge between rural and urban areas. Other interesting insights are provided by examining the interactions between factors, e.g., patients affected by comorbidities are more responsive to hospital quality even though they are distant.

**Conclusions:**

Reorganising healthcare services to concentrate them in high-volume hospitals emerged as a crucial issue more than forty years ago. Evidence suggests that concentration strategies guarantee better clinical performance. However, in healthcare systems in which patients are free to choose where to be treated, understanding patients’ behaviour and what drives them towards the most effective choice is of paramount importance. Our study builds on previous research that has already analysed factors influencing patients’ choices, and takes a step further to enlighten which factors drive patients to choose between a small or a big hospital (in terms of volume). The results could be used by decision makers to design the best concentration strategy.

## Background

Planning healthcare systems in the most accurate and appropriate way is a key element for their correct functioning with respect to both efficiency and effectiveness. For this reason, the interest in planning problems for healthcare systems has largely increased in recent years [1]. Planning is a multidimensional topic involving different hierarchical levels, from strategic (e.g., how many hospitals to have in a territory and their dimension) to operational (e.g., how many beds to have in a ward). Researchers have investigated different aspects of planning, such as decisions about the number of hospital services (such as beds or doctors) to be offered in a given region, their location [2], and details regarding the specific allocated resources [3, 4]. In this study, we focus on strategic planning decisions. Specifically, we deepen the dimension of healthcare structures in terms of volumes of activity, i.e., the number of interventions that will be performed in a set of hospitals (of a specific territory), with reference to a specific procedure.

When planning the number of interventions for a given procedure, concerns have been recently raised about the scattering of surgical interventions among a vast number of facilities due to the increasing amount of research documenting the risk of undermining patients’ health conditions [5–8]. This phenomenon has been called *volume-outcome association*, and it reports lower volumes of activity (i.e., the number of interventions performed by a facility) being associated with lower clinical outcomes (e.g., higher mortality rates, complication rates, etc.). The explanation for the effect originates from structural factors and professionals’ experience [9, 10]. In fact, outcomes may be related to the familiarity of staff, in particular the surgeon, with the treatment [11, 12], and with the processes for dealing with postoperative complications [6]. In Italy, the National Outcome Evaluation Program (*Programma Nazionale Esiti* - PNE) [13] documented the occurrence of the volume-outcome association for fourteen procedures using Italian national data [5, 14], thus confirming the existence of this relationship in current clinical practice. Building on this evidence, a debate has grown on the possibility of setting international standards for condition-specific volume thresholds to be respected worldwide [15].

These concerns about dimensioning have had an impact on the planning decisions adopted thus far [15], leading to the application of concentration policies. Such policies might imply different actions, such as those reported in the following. A first possibility is the adoption of the mentioned volume thresholds, which forces hospital wards to perform at least a minimum volume of activity [16]. A second alternative is the identification of hub and spoke hospitals in which interventions are primarily directed to hub hospitals [17]. Finally, wards can be closed to reduce the number of structures offering the same service in a given territory [18].

In general, the literature on strategic healthcare planning relies on tools such as models and algorithms [2, 19–21], and the model formulations are strictly related to the perspective of the considered actors. The actor perspective, in fact, implies a specific aim. Among the main stakeholders of the healthcare sector (e.g., decision-makers, patients, practitioners, etc.), the managerial literature often considers the perspective of the decision-maker, for whom efficiency (e.g., in terms of the number of beds or costs of the procedures) is usually the main objective. Few studies, instead, propose integrated approaches in which decision-maker and patient perspectives are included together in the decision-making process [22–24]. Including patient perspectives is particularly important in contexts in which patients are free to choose where to be treated, as their decision can influence the actual hospital volume.

The patient perspective is usually considered in the literature on health economics, which investigates what factors influence patient choice. From this literature, the main influencing factors are related to hospital and patient characteristics [25, 26]. Regarding hospital characteristics, the most influencing factors are distance and quality. Specifically, distance matters, with people preferring hospitals closer to their home, everything else being equal [27]. Quality is also important, with patients prepared to bypass their local provider if they believe quality is higher elsewhere. This holds irrespective of how quality is measured [28], whether by waiting times [29, 30], readmission rates [31], mortality rates [30, 32], and official rankings [33]. Distance and quality are still relevant in the decision process even when patient personal characteristics, such as age, social and clinical conditions, are considered [32, 33].

When considering patients’ roles, it is essential to understand their specific decision process. In this respect, several papers have documented patients’ decision process, specifically in the Italian context, in which patients have always been free to choose where to be treated, while this is not the case in other European countries (e.g., United Kingdom) [34]. Especially in the case of hospitalisation, for which Italian patients do not pay, hospital quality is likely to be an important determinant of individual choices [35], even though accurate information is hard to come by. In fact, unlike other countries, Italy has a brief history of publicly available hospital ranking, which refers to the PNE, which has been published from 2011 [13]. Moreover, PNE does not cover all the Italian hospitals for all the outcome indicators (as will be explained in the following), and the actual use made by citizens has not yet been documented.

Because of their freedom to choose, Italian patients may choose hospitals that do not respect the international standards of volumes of activities, notwithstanding the volume-outcome association. We can label these hospitals as *small* for the sake of simplicity, while we label *big hospitals* those that respect the international standards of volumes of activities. Our study begins from this rationale: since concentration (also called regionalisation or centralisation) strategies aim at guiding patients towards big hospitals that perform better [13, 36, 37], attention should be given to the factors that contribute to this goal. For this reason, we analyse which factors drive patients to choose big or small hospitals. Building on previous literature [38, 39], we consider the impact of patient characteristics, and two other elements. Specifically, we consider: (i) the characteristics of the closest big hospital (which should be perceived by patients as the most immediate to be chosen), and (ii) the territorial characteristics (i.e., characteristics of the set of hospitals among which the patient can choose, such as the number of small and big hospitals included in a geographical radius of distance). While the decision-maker cannot influence patient characteristics, territorial characteristics can be modified by new planning policies. In addition, the interaction between personal and territorial characteristics can be exploited to design more effective concentration strategies. Eventually, our results will shed light on the characteristics that might influence patients to choose big or small hospitals, providing some insights on how decision-makers can drive the latter to move to big hospitals. The analysis considers a case study in Piedmont (Italy) focusing on surgeries for colon cancer, one among the most frequent oncological pathologies in which, from the patients’ perspective, the continuity of care needed may interfere with the preference for quality.

 The remainder of the paper is organised as follows. A detailed description of the empirical context, data and methodology is given in Sect. 2. Section 3 presents the numerical results, while managerial insights and implications are discussed in Sect. 4, together with the limitations of the proposed study and ideas for future research.

## Methods

### Empirical context

Our study focuses on colon cancer surgery performed in the hospitals located in Piedmont, a region with 4.4 million inhabitants in northwest Italy. The focus on colon cancer is motivated by the combination of epidemiological, managerial and organisational considerations, related to: (i) its prevalence, (ii) the provision of a screening test that increases patients’ awareness of the disease, (iii) the continuity of care required by patients who extends patients’ journey, and (iv) the documented importance of the concentration of interventions in specialised hospitals to improve the patient survival. In fact, colon cancer represents, in Italy, the second most frequent oncological pathology: there were estimated 43,700 cases in 2020, 12% of all diagnosed cancers [40]. For patients diagnosed with colon cancer, a surgical intervention, called resection, is necessary to remove the cancer. After surgery, the patient receives periodic follow-ups by both oncologists and surgeons. This process results in cancer patients being included in personalised care pathways, which involve healthcare services pre-, during, and post-surgery occurrence. For this reason, they can become attached to a healthcare structure and eventually prefer to refer to it, giving priority to continuity in the trade-off with quality.

Colon cancer is among the clinical areas in which the volume-outcome association has been confirmed by the PNE Italian program [14]. International guidelines recommend that hospitals provide a minimum of 50 or 70 colon cancer surgeries per year [41]. However, there have been long-standing concerns that hospitals in Italy are undertaking small volumes of colon cancer surgery, the average number of colon surgeries performed by Italian hospitals in 2015 being 34 [42].

Italy’s healthcare system is a regionally based National Health Service (NHS), which provides universal coverage essentially free of charge at the point of delivery [43]. As already mentioned, in Italy, patients are free to choose the hospital to be treated in. For this reason, the study of the supply side (i.e., how hospitals should be organised) must include an analysis of the demand side (i.e., how patients choose hospitals). Regardless of the specific chosen hospital, in this paper, we focus on the difference between patients who choose hospitals performing larger or smaller volumes than the advocated threshold of 50 interventions per year. In fact, in the context of freedom of choice, the key message to be conveyed to patients should be to choose whatever hospital of sufficient quality. More specifically, the quality can be related to the mentioned volume threshold, as suggested by the international guidelines [41].

**Fig. 1:**
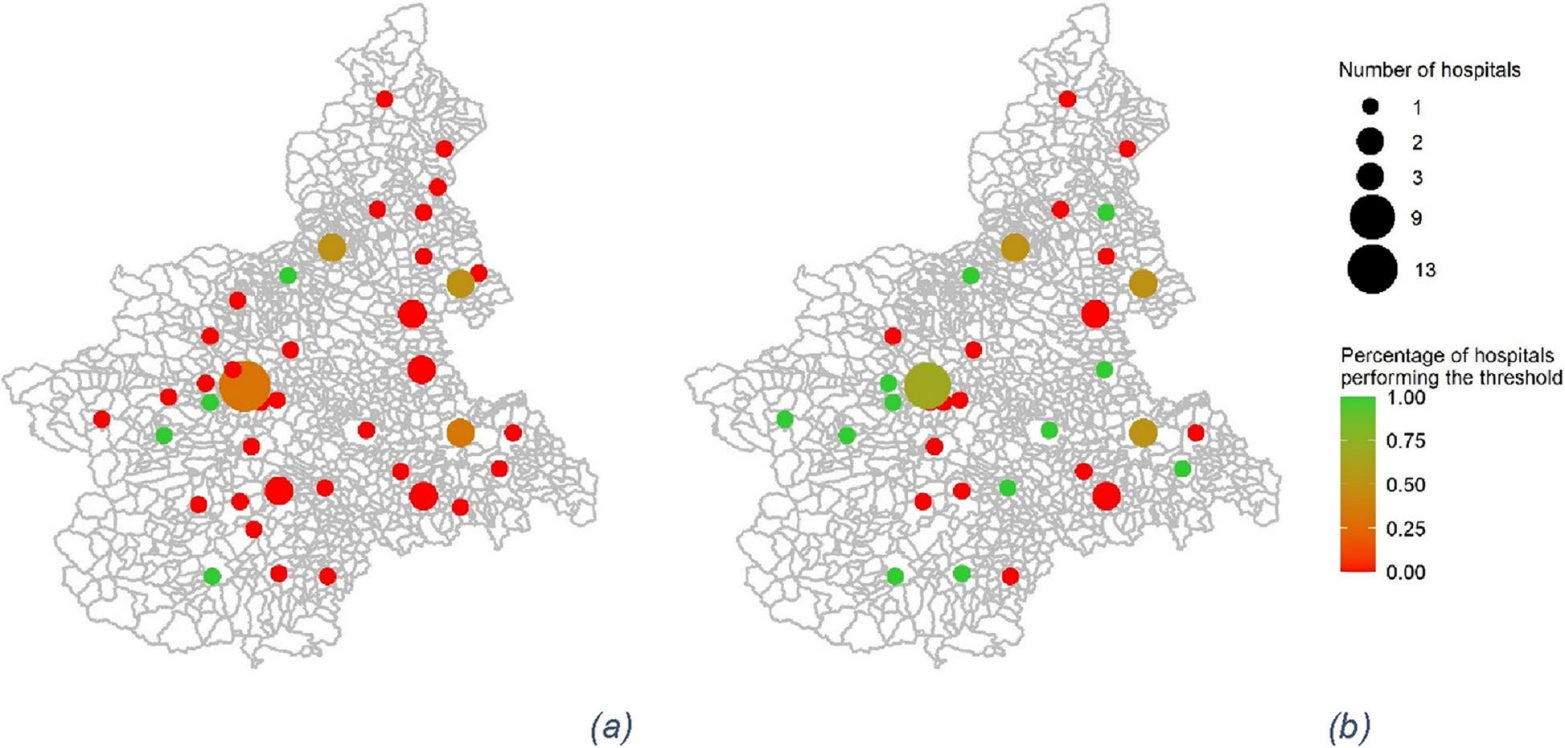
Maps of Piedmont showing hospitals performing colon cancer interventions in 2003 (**a**) and 2018 (Maps of Piedmont showing performing colon cancer interventions in 2003 (**a**) and 2018 (**b**). Figure created by authors using R (Rstudi). Figure created by authors using R (Rstudio 2022.02.0 + 443)

The maps of Piedmont in Fig. 1 depict the distribution of hospitals (dots) performing colon cancer interventions in 2003 (Fig. 1a) and 2018 (Fig. 1b). The size of the dots expresses the number of hospitals in the same city, while the colour represents either if the hospital has performed more than 50 interventions per year (in the case of one hospital per city) or the percentage of hospitals that did it (in the case of multiple hospitals per city). In some areas more than one hospital provides colon cancer surgery, and there are also several hospitals operating in nearby cities. Moreover, a reduction occurred between 2003 and 2018 in the number of hospitals in Piedmont performing colon cancer interventions, possibly related to policy decisions or organisational needs. Overall, the number of hospitals decreased from 61 to 46. This reduction led to a change in hospital volumes during the same period, with the mean volume increasing from 35 to 52, and the median volume increasing from 27 to 47. Indeed, the number of green dots, identifying the hospitals performing more than 50 interventions, increased from 2003 to 2018. The Piedmont region has also structured an oncological network composed of healthcare structures dedicated to screening, diagnosing and follow-up of colon cancer cases. In 2016, a step forward was made by identifying specific hospitals for each geographical area for surgery [44]. All these elements make colon cancer in Piedmont an interesting context to study patient distribution among hospitals over the years.

### Data source and study design

Our study is a retrospective cohort study [45]. To build the patients’ dataset (unit of analysis: the patient), we started from routine administrative patient-level data of the Hospital Discharge Database of Piedmont Region, a database that contains records of all the episodes of hospitalisation. We identified patients belonging to the cohort by selecting all the Italian residents in Piedmont who were admitted to hospitals located in Piedmont between January 2004 and December 2018, and received an intervention for colon cancer surgery (identified through the codes of the International Classification of Diseases – 9th revision, in particular codes for diagnosis 153, 197.5, codes for intervention 45.7, 45.8, 45.9, 46.03, 46.04, 46.1). We thus excluded patients being treated in urgent conditions and we included only elective hospital admissions, as elective admissions might imply a thoughtful choice by the patient. We omitted patients younger than 18 years and we excluded patients who went to private hospitals, because additional elements (e.g., costs) informed their choice. Data were not available for patients resident in Piedmont and treated in hospitals outside the region. From the Hospital Discharge Database we also retrieved personal and clinical data about each patient, including their residence.

To associate to each patient the information related to his/her hospital choice, we created another dataset containing hospital information, i.e., the hospital dataset (unit of analysis: the hospital). To do this, we aggregated all the patients’ records at hospital level, to summarise some hospital characteristics (such as their median waiting time, as will be described in the following section). Based on the unique identification code, we also linked to each hospital: (i) the information published by the Ministry of Health about its geographical location, and (ii) the information published by the PNE about its performance. It should be noted that, even if the PNE is based on all Italian hospitalisation records and thus it considers all the Italian hospitals, it does not contain information on the adjusted mortality rate for all the hospitals performing colon cancer interventions. This lack is due to methodological reasons, similarly to other papers in the literature [31]: the risk adjustment technique requires a minimum sample size for its application. Moreover, data on mortality from PNE is only available since the program started, in 2011. For this reason, models with and without adjusted mortality rate will be considered in the following. Once the hospital dataset was created, we used it to feed the patient dataset with hospital information, by creating some of the variables that will be described in the following section.

### Variables

The dependent variable for the study is a boolean variable, which has a value of 1 if the patient has chosen to be treated in a hospital that, during the year before the treatment, has performed more than the threshold of 50 interventions (i.e., a big hospital), and 0 otherwise (i.e., a small hospital). We call this variable *big*.

Our aim is to understand how this variable is affected by three groups of independent variables, i.e., “personal” variables, “closest big hospital” variables, and “contextual” variables. The first group of variables refers to patients; the second is related to the closest hospital that performs more interventions than the threshold, i.e., the hospital that patients should reckon as the most immediate location to be treated; the third refers to the context, and it is composed of hospitals within a certain distance from the patients’ residence. For all the variables, we built on the information reported by the literature to identify the most relevant aspects to be analysed.

Regarding personal variables, we considered age, gender, comorbidity, and geographical area. As an indicator of comorbidity, we use the Elixhauser index [46] and we categorised it into two classes, i.e., zero or one comorbidity vs. more than one comorbidity. Regarding the geographical area, we associated to each of the municipality of residence with the classification made by the Ministry of Economic Development, which categorises cities into 6 main groups, ranging from urban (1–3) to rural (4–6) locations. We used a boolean variable with a value of 0 for groups 1–3, and a value of 1 for groups 4–6.

The second group of variables, related to the closest big hospital, includes distance, waiting time and mortality rate. The distance of each patient to the closest big hospital was defined as the fastest route by car from the patient’s home to the hospital calculated in kilometres [47]. The patients’ home was considered the centroid of the patients’ municipality of residence, and the centroid of the hospital municipality was considered for its location. The big hospital waiting times have been calculated as the median of waiting times for patients treated in that hospital, and patient’s waiting times are measured as the time (in days) from the day the hospital specialist adds the patient to the waiting list to the day the patient is admitted (both information are contained in the Hospital Discharge Database). For big hospital mortality rates, we used, for each hospital and for each year, the official adjusted mortality rate published yearly by PNE, for all the years in which the program has been active, i.e., since 2011. For both waiting times and mortality rates, we lagged the variables by one year, to ensure that patients had the possibility of becoming aware of this information. If a patient had more than one big hospital at the same distance, we built the closest big hospital variables by taking the best performance measures. For distance, it is the same among the equally closest big hospitals; as for the waiting times and mortality rates, we individually considered the lowest values among those of the closest big hospitals.

The third group of variables (i.e., the contextual variables) consists of two variables related to the number of small hospitals (i.e., performing less than the threshold of 50 interventions) and to the number of big hospitals (i.e., performing more than 50 interventions) within a radius of 10 km from patients’ residence. As the length of the radius impacts the number of small hospitals included in the variable, its value has been tested through a sensitivity analysis with values of 30 and 50 km.

All the variables were calculated (e.g., the contextual variables) and/or attributed (e.g., the personal variables) for each patient. In this way, the changes that occurred over time in hospitals’ performance or territorial configurations were embedded in the variables of the second and third groups, since each patient is associated with the information collected in the year (or the year before) he/she was treated.

### Methodology

First, a set of descriptive statistics is computed on the patients’ dataset, to identify the main characteristics of the studied cohort, and to map the existing differences in the choice of big or small hospitals depending on the independent variables. Hence, personal variables, big hospital variables and contextual variables are summarised (with the mean in the case of continuous variables, percentage in the case of categorical variables) both for all the patients, and for the patients that went to a small or a big hospital. The p-value of the statistical tests for the difference between the values of the two groups of patients (those who went to a small and those who went to a big hospital) are calculated (Welch Two Sample t-test and Wilcox test for continuous variables, Pearson’s Chi-squared test for categorical variables) Second, the logistic regression model is run on the patients’ dataset, to enlighten the direction and the magnitude of the influence of the independent variables on the probability of choosing a big hospital. More specifically, four regressions are run: model (1) represents the first baseline case that includes the personal variables, the big hospitals variables and the contextual factors; model (2) provides the results with also the coefficients of the interactions with individual-specific variables, which provide insights about specific types of patients; models (3) and (4) repeat the analysis only for the cases in which data on mortality rate are available, i.e., during years 2012–2017 for hospitals for which the PNE has published the mortality indicator. Interaction terms will allow to draw conclusions about the interplay of “personal”, “closest big hospital” and “contextual” variables. Year fixed effects are included in all regressions, to account for changes that occurred during the observed period and are related to external factors. Estimates will be reported along with the 95% confidence intervals (CI). All statistical analyses were performed with R (Rstudio 2022.02.0 + 443).

## Results

**Fig. 2:**
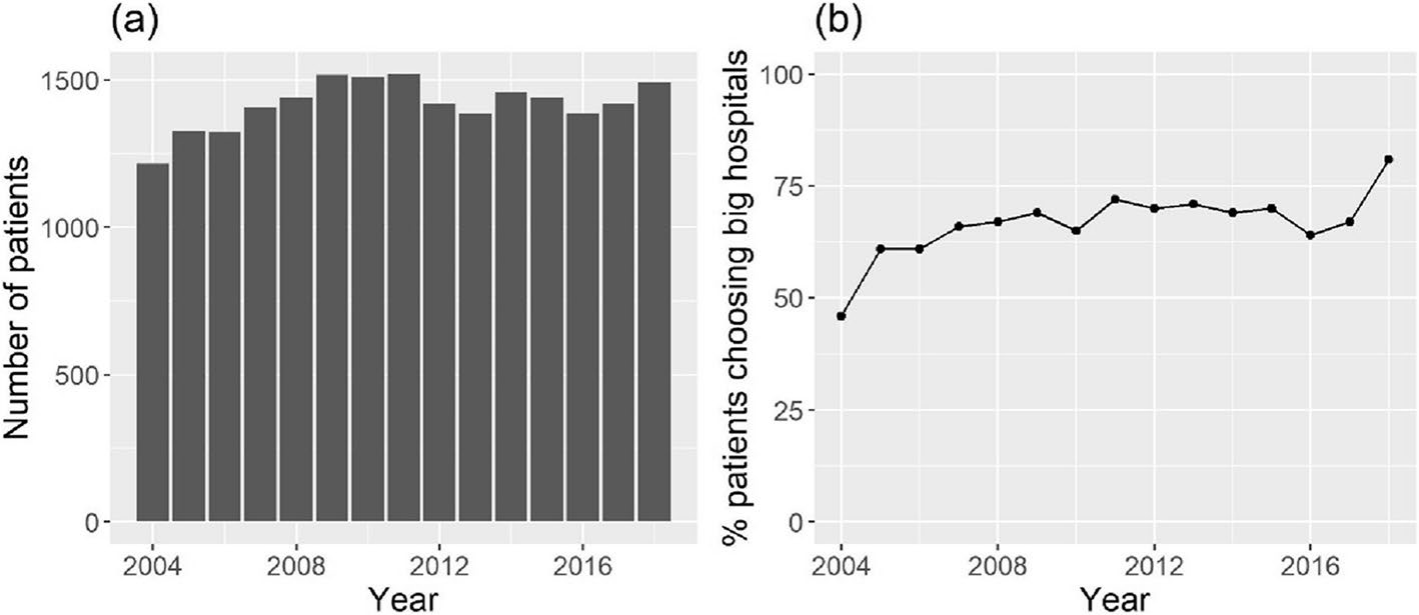
**a** Annual number of colon cancer interventions from 2004 to 2018 in Piedmont, Italy. **b** Percentage of patients having colon cancer interventions in Piedmont, Italy, going to hospitals that perform more than the threshold

The sample is composed of 33,222 patients receiving colon cancer interventions in Piedmont, Italy, from 2004 to 2018. The annual number of interventions is shown in Fig. 2a, while Fig. 2b reports the percentage of patients going to hospitals that perform more than the threshold, which is on average 67%.

Table 1 describes the main characteristics of the cohort related to the three groups of considered independent variables. As said, each variable is analysed both for all the patients (third column), and for the patients who went to a small (fourth column) or a big (fifth column) hospital. The last column reports the p-value of the statistical tests for the difference between the values of the fourth and fifth columns. For the complete cohort (column All patients), the mean age of patients is 71 years, 46% are females, 47% of patients have more than one comorbidity, and 15% live in a rural area. For the characteristics of the closest big hospital, the distance varies from 0 km (1st quantile) to 24 km (3rd quantile), with a mean value of 14.8 km (median value 12.1 km). The waiting time is on average 16 days (median 13 days, 1st quantile 10 days and 3rd quantile 19 days), and the mortality rate 3.7% (median 2.7%, 1st quantile 1.4% and 3rd quantile 5.2%). Moreover, patients have, on average, a choice of 1.9 small hospitals (median 1, 1st quantile 0 and 3rd quantile 2) and 1.6 big hospitals (median 0, 1st quantile 0 and 3rd quantile 2) within a radius of 10 km.

**Table 1:** Main characteristics of the cohort: patients receiving colon cancer intervention in Piedmont, Italy, from 2004 to 2018

Group	Name	All patients	Patients going to small hosp	Patients going to big hosp	p-value
Personal variables	Age (mean years)	70.5	71.0	70.2	< 0.001
	Sex (% female)	45.8	44.9	46.0	0.07
	Comorbidity (% having more than 1)	47.14	42.47	49.44	< 0.001
	Rural (%)	14.5	19.7	11.9	<0.001
Big hospital variables	Distance (mean km)	14.8	21.8	11.3	<0.001
	Waiting time (mean days)	16.0	15.9	16.1	0.13
	Mortality rate (mean %)	3.7	4.0	3.6	<0.001
Contextual variables	Number of small hospitals	1.9	1.5	2.1	<0.001
	Number of big hospitals	1.6	1.0	1.9	<0.001

From the table, some differences emerge between patients who went to a big hospital and those who chose a small hospital. Patients who have been treated in a small hospital are slightly older, with a lower number of comorbidities, and more often come from rural areas. Moreover, they are more distant from big hospitals, and their closest big hospitals have higher mortality rates. Additionally, these patients have fewer small hospitals but also less big hospitals within the radius of 10 km from their residence. This combination of characteristics makes important to consider them and their interaction when examining the choice of big or small hospitals, which can be done with a multivariate logistic regression.

The results of the performed regressions are shown in Table 2. Four regressions (indicated as Models (1)-(2)-(3)-(4)) are presented in the table and will be discussed in detail in the following. For all the regression models, since we include interactions with personal characteristics, the baseline coefficients are related to a reference patient, who is male, 72 years old, lives in an urban area, and has 0 or 1 comorbidity.

**Table 2:** Results from regression models applied to the cohort of patients receiving colon cancer intervention in Piedmont, Italy, from 2004 to 2018. *p < 0.05; **p < 0.01; ***p < 0.001

			Without mortality rate		With mortality rate	
			Model (1)	Model (2)	Model (3)	Model (4)
Personal variables	**Sex**		0.046	0.182	0.068	0.057
	**Age**		-0.012***	0.0004	-0.013***	0.015
	**Comorbidity**		0.349***	0.458***	0.361***	0.697***
	**Rural**		0.148**	-0.479***	0.088	-0.697**
Big hospital variables	**Distance**		-0.049***	-0.06***	-0.056***	-0.062***
	**Waiting times**		0.005**	0.02**	-0.009**	-0.006
	**Mortality rate**				0.008	-0.006
Contextual variables	**N small hospitals**		-0.116***	-0.034	-0.118***	-0.047
	**N big hospitals**		0.128***	0.060	0.086**	0.051
Interactions terms	Distance*	**Age**		-0.0004***		-0.001***
		**Sex**		0.0005		-0.003
		**Comorbidity**		0.004		-0.003
		**Rural**		0.032***		0.039***
	Waiting times*	**Age**		-0.0003		-0.001*
		**Sex**		-0.006		-0.001
		**Comorbidity**		-0.009**		0.004
		**Rural**		-0.012**		-0.005
	Mortality rate*	**Age**				0.002
		**Sex**				0.034
		**Comorbidity**				-0.075***
		**Rural**				-0.025
	Number of small hospitals*	**Age**		-0.001		-0.0003
		**Sex**		-0.033		-0.024
		**Comorbidity**		0.002		0.013
		**Rural**		-0.390***		-0.573***
	Number of big hospitals*	**Age**		0.001		-0.004
		**Sex**		0.011		-0.0001
		**Comorbidity**		-0.039		-0.089
		**Rural**		1.262***		1.062***

From models (1) and (3), we see that personal characteristics indeed play a role. Older patients have a lower probability of going to big hospitals, while the opposite occurs for patients having more than one comorbidity and living in rural areas. For the big hospital characteristics, distance from the closest big hospital matters, since the longer the distance, the lower the probability of choosing a big hospital. Waiting times, instead, show opposite coefficients in the two models, and the variable for mortality rate does not appear to be significant. About contextual variables, both the variables counting the number of small and big hospitals within 10 km are significant and show opposite sign coefficients: the higher the number of close small hospitals, the lower the probability of going to a big hospital, while the opposite happens if the number of close big hospitals increases. Overall, even if the sample size is reduced from models (1) to (3), results on common variables remain very similar.

**Fig. 3:**
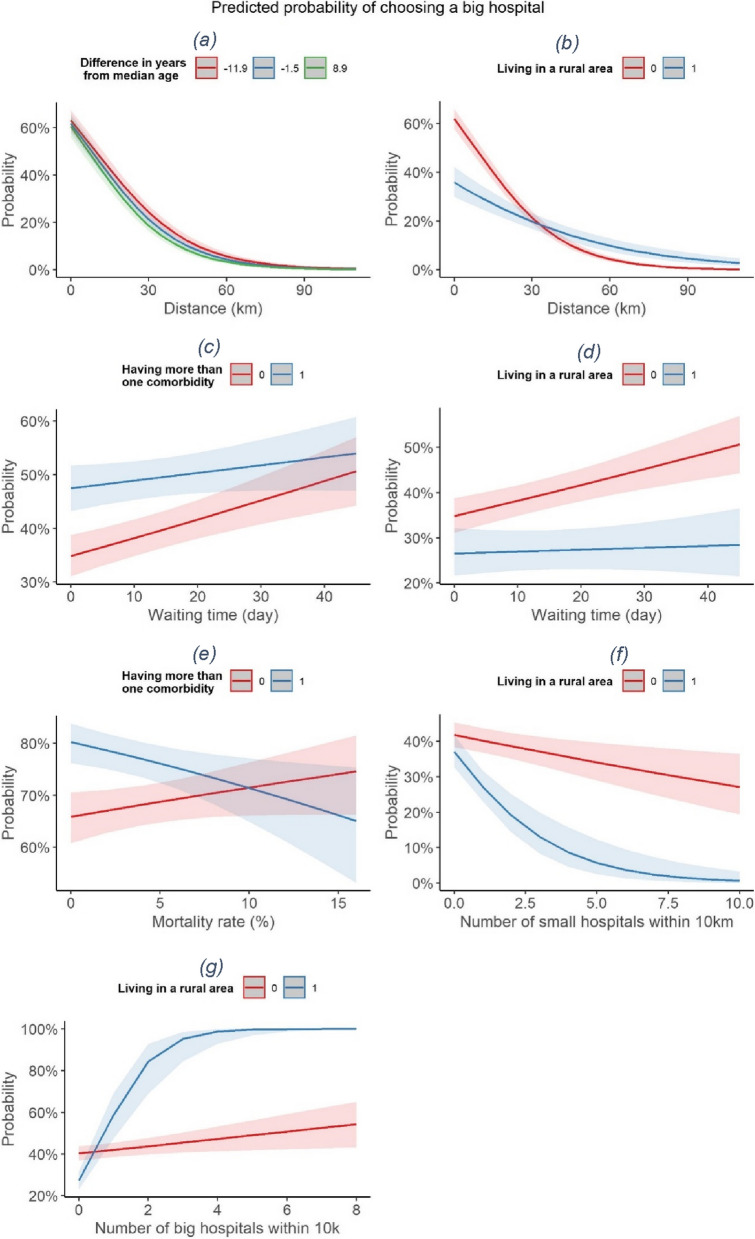
Predicted probability of choosing a big hospital characterised for various significant interaction terms. Cohort: patients receiving colon cancer intervention in Piedmont, Italy, from 2004 to 2018

The interpretation of the results partially changes when adding the interaction terms in models (2) and (4). The variable *age* is no longer significant, while the variable *rural* has a negative coefficient. This change confirms the importance of inserting the interaction terms, which help to highlight the complex interplay existing among factors. Figure 3 supports a complete interpretation of all the coefficients whose interaction terms are statistically significant.

A significant interaction exists between the variable *distance*, i.e., the distance from the closest big hospital, and both age (Fig. 3a) and rural conditions (Fig. 3b). In particular, the negative impact of distance is emphasised by age, and this effect can also be observed when looking at how the impact of distance changes for patients living in rural vs. urban conditions. In fact, rural patients have a lower predicted probability of choosing a big hospital, but this probability decreases slowly with distance compared to what happens to patients living in urban areas. A possible explanation is that patients from rural areas are more used to travel in general, and thus they perceive less the disutility caused by distance.

The occurrence of more than one comorbidity changes as well the choice of being treated in big hospitals. In fact, patients with more severe health conditions have a higher probability of choosing a big hospital, as shown by the positive and significant comorbidity coefficient in all the models. In addition, they reveal a preference for lower waiting times, as Fig. 3c shows. Also, patients with comorbidities tend to choose big hospitals with lower associated mortality rates, as displayed in Fig. 3e. Indeed, these patients might be willing to receive the best care in the shortest time, because of their more complicated clinical conditions.

A result that needs to be further investigated is related to the interaction between waiting times and rural conditions (Fig. 3d). It seems that patients living in urban areas increase their probability of choosing a big hospital when the closest big hospital has higher waiting times. This result seems in contrast with the literature documenting that patients dislike waiting. We may suppose that, in the urban context, waiting times are known to be higher in general, and the results are a proxy for the reputation of the hospital since they are caused by a higher number of people asking to be treated there.

Eventually, contextual variables are particularly important for patients living in rural areas (Fig. 3f − g). In general, an increase in the number of small hospitals leads to a decrease in the probability of choosing a big hospital, and the increase in the number of big hospitals leads to an increase in the probability of choosing a big hospital. For urban patients increase and decrease are both gradual, while they are not for rural patients. In fact, for rural patients, when there are more than two close small hospitals, the percentage of patients choosing a big hospital drastically decreases. However, when there are more than two big hospitals, the percentage of patients choosing them sharply increases.

## Discussion

Reorganising healthcare services to concentrate them in high-volume hospitals emerged as a crucial issue more than forty years ago [48] and still dominates the international debate [15, 36, 37]. Notwithstanding the complexity of the factors affecting planning healthcare systems, evidence suggests that regionalisation and concentration strategies guarantee better clinical performance [49, 50]. To this aim, multiple actions have been put into place.

However, differences arise between healthcare systems based on the possibility that patients have or do not have to choose where to be treated. In Italy, as in other countries, the latest decision rests on patients, and this implies that the allocation of volumes among hospitals ultimately depends on their behaviour, which hence cannot be neglected [22].

Given these elements, understanding patients’ behaviour and what drives them towards the most effective choice is of paramount importance. Previous studies in the literature have already analysed the factors influencing patients’ choice (e.g., distance, quality) [28, 32, 33]. Our study takes a step further to enlighten which factors influence patients to choose between a small or a big hospital (in terms of volume).

First, our results confirm that personal characteristics play a primary role in the patient decision-making process. Indeed, older patients appear the most likely to choose small hospitals: the older the patient is, the larger the decrease in the probability of choosing a big hospital if it is far from the patient’s residence, especially for patients living in urban areas. Such a decrease is smoother for rural patients. Hence, policy makers should pay attention to the population located in the geographical area where the hospital is located. However, the interplay documented among personal characteristics, hospitals, and contextual features calls for the need to consider them together.

Hospital characteristics have an important influence on patient choice: distance is confirmed to have a negative influence, while waiting times and mortality rates are difficult to evaluate on their own, and interactions with other factors must be considered. A relevant result emerges from the variable of comorbidity: patients affected by comorbidities have a higher probability of choosing a big hospital if it has a better performance than the closer small hospitals. The increased awareness of these patients may also be due to the tighter follow-up they have to receive based on their more severe health conditions.

Moreover, the results from contextual variables support the importance of closing small hospitals, especially when they are close to big hospitals, even if the interaction terms explain which categories of patients are more influenced by their presence. Small hospitals could, in fact, attract patients who have big hospitals in the same area, regardless of the quality offered by them, especially for urban and older people with no critical health conditions. This should guide policy makers to apply different concentration policies in rural and urban areas.

The recommendation for small hospitals to cease performing surgical interventions does not automatically force them to close, especially for clinical areas such as colon cancer surgery. The diagnosis of colon cancer directs patients to undertake a care pathway composed of multiple steps, ranging from surgery to follow-up, which can be structured with a territorial configuration of hub and spoke. While the learning curve advocated for surgery should be set as a target for hubs, small hospitals can indeed play a key role as spokes that specialise in other functions for whom geographical and social proximity to patients becomes crucial. To contribute to this change, resources should be dedicated to Transitional Care, i.e., the set of actions designed to ensure coordination and continuity among different care levels and settings within the same structure or among different organisational structures [51]. Nonetheless, in the Italian context, the situation appears varying and without a framework of reference [52].

Our study has strengths and limitations. We consider, as the added main value, our immediate research question on what drives patients’ choice. Even though the same question has also characterised other studies, we simplified the study of patients’ alternatives into a dichotomy between big and small hospitals, partitioned by the threshold of fifty interventions per year. Building on the scientific evidence that documents the clinical impact of overcoming this threshold, we believe that guiding patients towards big hospitals may well represent the policymaker’s ultimate objective. In fact, given that patients can choose whatever hospital, it is more relevant to guide their choice towards any high-volume hospital compared to a specific hospital.

Most studies in the literature have used methodologies such as choice models [27, 28, 53], agent-based simulation [54], surveys [55, 56], or multi-criteria decision-making methods [57]. In our study, the formulation of the dichotomy between big and small hospitals drove us to use the logistic regression. We believe that the lower complexity of this methodology matches well with the aim of simplifying the research question, which, formulated with the mentioned dichotomy of big vs. small hospitals, is immediately understandable by policy makers.

Finally, the strength of the immediate research question can also be read as a limitation when we think of the complexity of factors impacting hospital quality – which goes beyond attention to the volume alone [49]. Our choice has been to favour the use of volume thresholds as a proxy for quality, but results must be presented knowing that it is only part of reality [28, 41]. As such, the several changes that occurred over the long time period of the analysis, such as the change in prevalence, surgery volumes, local policies, and screening tests, need to be further investigated in the case of implementation of new health policies that start from our results. For the same reason, the decision on the closure of small hospitals in a territory should also consider other characteristics of the hospitals that may vary independently from the big/small feature (e.g., number of beds, other outcome measures, etc.). This may guide policy makers to hospital-specific decision strategies. Among the other limitations, our study is built on administrative datasets, limiting the amount of available patient information. For this, we included only a small set of personal characteristics, even though they are the ones documented to be more influential. A similar issue about data applies to the adjusted mortality rates that are not published by the PNE: we argue that the picture that we are losing concerns low number of patients. Furthermore, even though previous studies have argued that Italian patients, when choosing where to be treated, may use informal information (gathered either via social interaction [35] or their networks [58]) about hospital quality, the data used in our study did not allow to consider social interactions, word of mouth and/or the impact of the general practitioner’s referral. In this respect, future research should also extend the analysis to non-Italian citizens, for whom different referral dynamics may apply, related, for instance, to the presence of other patients with the same nationality having been previously treated. Despite these limitations, we believe that our research may support the international debate to design operational strategies that build on these findings.

## Conclusions

Within the debate on the concentration of healthcare services in high-volume hospitals, attention is needed to understand patients’ behaviour when patients are free to choose where to be treated. Our study builds on previous research that has analysed the factors influencing patients’ choice and takes a step further to enlighten which factors influence patients to choose between a small or a big hospital (in terms of volume). The results confirm the importance of personal characteristics such as age, hospital characteristics such as distance and contextual characteristics such as the number of small hospitals in the territory. Moreover, the analysis including interaction terms shed some light on the interplay occurring among factors. Overall, the simplification of the study of patients’ alternatives into a dichotomy between big and small hospitals (using the internationally advocated standards for the volumes of activity) provides results that are immediately understandable by policy makers and support them in guiding patients towards any high-volume hospital.

## Data Availability

The data that support the findings of this study are available from the Piedmont region but restrictions apply to the availability of these data, which were used under licence for the current study, and are not publicly available.
